# Basic concepts of cancer genetics and receptor tyrosine kinase inhibition for pharmacists. A narrative review

**DOI:** 10.1177/10781552231153814

**Published:** 2023-02-03

**Authors:** Kathleen B. Orrico

**Affiliations:** 43166 University of California San Francisco School of Pharmacy, Palo Alto, Menlo Park, CA 94025, USA

**Keywords:** Receptor tyrosine kinase, pharmacogenomics, tyrosine kinase inhibitors, molecular diagnostics, cancer

## Abstract

The intent of this review is to present basic genetic concepts key to understanding oncogenesis and the role receptor tyrosine kinase (RTK) inhibition plays in the targeted treatment of many cancer types. Oncogenic signaling by RTKs can result from genetic events such as point mutations, chromosomal rearrangements, structural variation, and gene amplification in the cancer genome. The cancer pharmacogenes discussed encode RTKs that exemplify the link between gene variation, the oncogenic process, and the basis of targeted approaches to treatment. Biochemical pathways often involved in oncogenesis and affected by RTK variation are reviewed. Molecular diagnostic testing for the presence of specific gene variants, alterations, and amplifications direct therapy to indicated tyrosine kinase inhibitors and monoclonal antibody drugs. As pharmacists are integral to the selection, preparation, and monitoring of chemotherapy, it is important that they understand the genetic basis for targeted therapies as well as the underlying disease process.

Cancer is a genetic disease and whether inherited or acquired, all cancer cells have at least one causal genetic alteration that leads to uncontrolled cell proliferation. Cancer-causing gene alterations can result in the dysregulation of normal cell replication and lifespan by disrupting the expression of genes encoding for proteins involved in the biochemical pathways that regulate cell division or participate in deoxyribonucleic acid (DNA) repair mechanisms. When corrupted, these signaling pathways can become oncogenic and foster a state of genomic instability, defined as an increased tendency to acquire further genetic mutations that promote tumor growth and spread.^1^ Genomic instability can also result from epigenetic factors that interact with the DNA nucleotide sequence to silence or turn on gene expression that is important to signaling pathways.^2^ Traditional chemotherapy drugs kill or stop cancer cells from dividing through various cytotoxic mechanisms.^3^ Newer more molecularly directed or targeted antineoplastic agents, such as the tyrosine kinase inhibitors (TKIs), interrupt the consequences or phenotype of mutated gene expression that is the genesis of unchecked cell proliferation and the oncogenic process.

As pharmacists are integral to the selection, preparation, and monitoring of chemotherapy, it is important to understand the genetic basis for targeted therapies as well as the underlying disease process. Tyrosine kinase inhibitors (TKIs) belong to an ever-growing class of antineoplastic agents available as small-molecule oral agents as well as injectable monoclonal antibodies. The intent of this review is to present basic genetic concepts key to understanding oncogenesis and the role tyrosine kinase inhibition plays in the targeted treatment of many cancer types.^4^ In particular, the focus will be on the drugs that inhibit the activity of receptor tyrosine kinases (RTKs) that are transmembrane signaling receptors located on the surface of cells. Biochemical pathways often involved in oncogenesis and affected by RTK variation will be reviewed as well as the very important cancer-associated genes that encode specific RTKs. As background, consider reading Basic Concepts in Genetics and Pharmacogenomics for Pharmacists (Orrico KB) available https://journals.sagepub.com/doi/full/10.1177/1177392819886875.^5^

## Malignant transformation

Progress in the ability to sequence and elucidate the expression of human genes has led to new paradigms for diagnosing, classifying, and treating cancer. To understand the pharmacodynamics and indications of targeted agents, including drugs that inhibit the activity of RTKs, it is helpful to explore the underlying disease process and the role genetic mutations play in oncogenesis.

Each person's cancer is genetically unique and can further evolve as a mass of cancer cells proliferates and spreads. As cancer cells replicate they continue to acquire genetic mutations and develop genomes that increasingly differ from that of the cell(s) from which they arose. As the process continues, tumor heterogeneity can occur when cells within the primary cancer as well as secondary clusters, take on new advantageous alterations and differ genetically from each other.^6^ Identifying specific genetic alterations through tumor molecular profiling throughout the disease course, informs the selection of effective targeted agents as well as the detection of drug resistance.

The initial triggering event that causes a cell to undergo proliferation, truncated differentiation, and impeded apoptosis, is not completely understood. What is known is that progression to fully aggressive, invasive cancer from a single aberrant cell typically involves a combination or series of molecular alterations occurring in different genes.^7^ Often these acquired genetic alterations foster the survival of the proliferating tumor mass such as establishing an adequate blood supply through angiogenesis or evading immune system surveillance.

## Cancer-associated genes

Somatic genes that are commonly involved in oncogenesis and tumor progression can be categorized into two types. The first type called proto-oncogenes produce proteins such as growth factors or enzymes that participate in the network of signaling and biocellular pathways that control cell division, inhibit cell differentiation, or halt normal cell death (apoptosis). If the expression of a proto-oncogene becomes overactivated through mutation or gene amplification (multiple gene copies), it is referred to as an oncogene.^8^ Oncogenes can promote unregulated cell division at abnormal times or in abnormal cell types, fostering transformation to a proliferating clone of cancer cells.

Tumor suppressor genes belong to a second category of genes that are commonly altered in the genomes of cancer cells. Unlike proto-oncogenes that lead to transformation when they experience a gain-of-function alteration, tumor suppressor genes contribute to oncogenesis when typically both gene copies become inactivated or experience a loss of function. Tumor suppressor genes encode for quality control proteins that are often involved in normal cell cycle checkpoints and DNA repair mechanisms and if corrupted by mutation or inactivation, can lead to oncogenesis.^9^ Identifying cancer-associated gene variants and other alterations common to cancer cell genomes has been the goal of several large research endeavors including The Cancer Genome Atlas Project (TCGA).

Launched in 2006, the TCGA used genome sequencing and bioinformatics to analyze over 20,000 rigorously collected primary tumor tissue samples from 33 different cancer types and compared them with matched, normal tissue samples.^10^ As new sequencing and research techniques were developed, TCGA extended molecular profiling beyond nucleotide sequencing to include data about the transcriptome, which captures the expression of ribonucleic acids (RNAs); the epigenetic profile, which identifies DNA and histone modifications such as methylation and acetylation; as well as characterization of the proteins produced. The result is a catalog or data set of cancer genetic information made accessible to researchers around the world for further exploration and drug development.

One such 2018 analysis of 9125 tumor samples profiled by TCGA (Sanchez-Vega F *et al*.) found that 89% of tumors from the 33 cancer types expressed at least one driver genetic alteration in one of 10 cellular signaling pathways.^11^ Driver gene mutations typically encode for altered proteins that abnormally move signaling pathways forward. The study also found that 57% of the tumor samples contained at least one alteration targetable with an available drug and 30% contained two or more potentially targetable alterations. Several of the identified altered genes encoded tyrosine kinase enzymes that regulate the activity of oncogenic pathways, the cell cycle, and the complex interplay between the two.

One important oncogenic pathway identified in the TCGA study as being frequently altered in cancer is the mitogen-activated protein kinase/extracellular signal-regulated kinase (MAPK/ERK) signal transduction pathway (also known as the RTK-RAS-RAF-MAPK). The MAPK/ERK pathway consists of a cascade of kinases and other proteins that induce mitosis by relaying signals regulating various cellular processes, including proliferation, from the surface of a cell into the nucleus.^12^ The signal is passed through the cell cytoplasm as a chain of phosphorylation events involving kinases, which ultimately activate transcription factors within the nucleus to prompt mitogenic gene expression.

**Figure 1.**
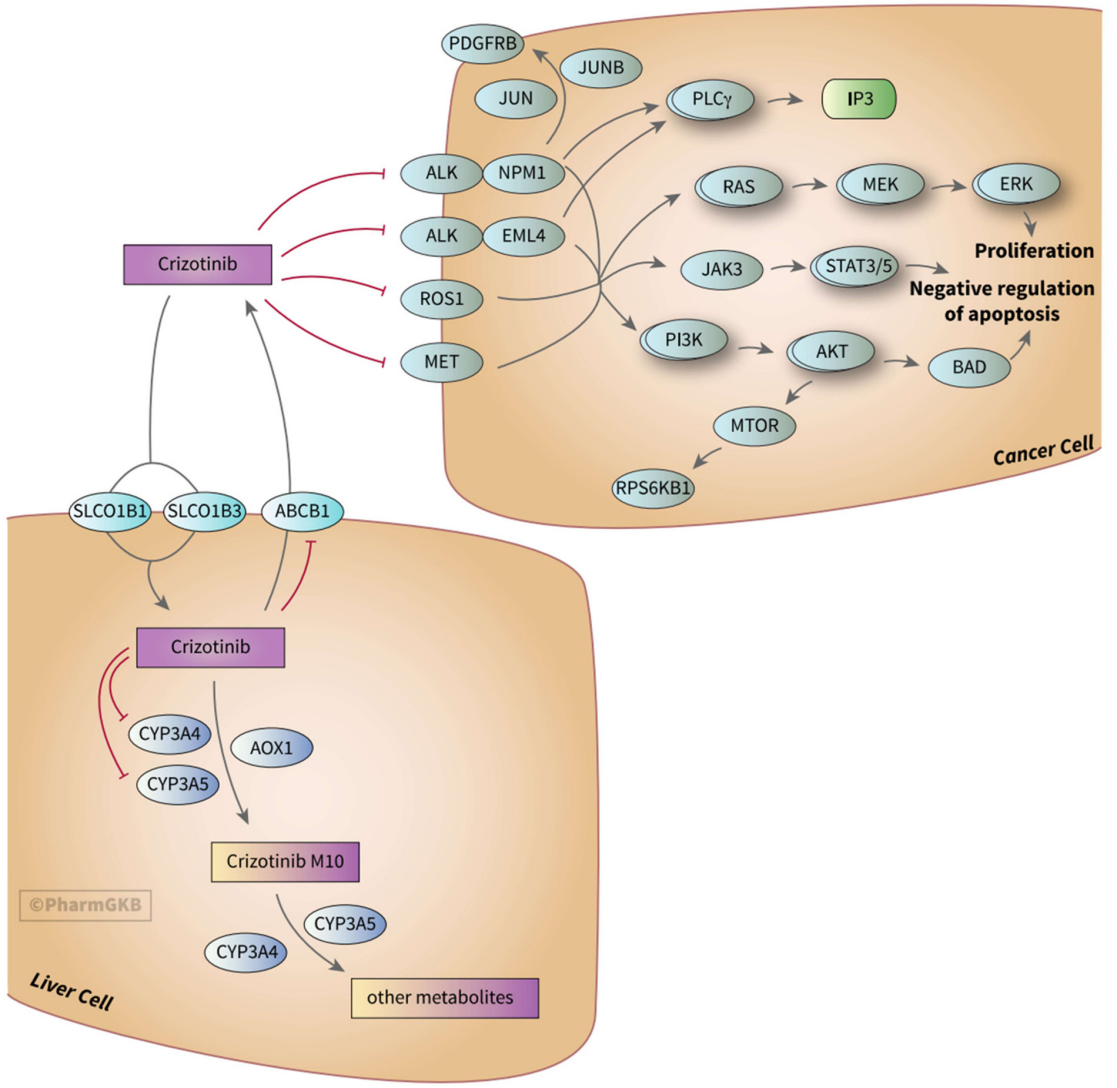
Crizotinib Pathway, Pharmacokinetics/Pharmacodynamics.

Source: PharmGKB Accessed 1/24/2023 https://www.pharmgkb.org/pathway/PA166178009

Another oncogenic signaling pathway identified in the study as being overactive in many different cancer types is the phosphoinositide-3-kinase–protein kinase B/Akt (PI3K-PKB/Akt) also known as the PI3K/Akt cell survival or pro-growth pathway.^13^ In response to conditions in the cellular environment, PI3K/Akt transduces a signal that can progress the cell cycle forward, stimulate nuclear gene transcription to increase the production of proteins and glucose needed to metabolically support increased cell growth, and ultimately influence cell survival. The PI3K/Akt pathway also participates in “cross-talk” or integration with other downstream signaling pathways including MAPK/ERK. Figure 1 offers a depiction of how the drug crizotinib can inhibit receptor signaling at the cell surface and reduce signal transduction to additional intersecting pathways including the Janus kinase signal transducer and activator of transcription (JAK-STAT) pathway that regulates apoptosis, as well as the phospholipase C gamma (PLC-gamma) pathway that is involved in cell proliferation.

## Kinases and tyrosine kinase inhibitors

Overall greater than 50% of identified proto-oncogenes produce proteins with kinase enzyme activity that often function as driver components of signaling pathways.^14^ A kinase is an enzyme that phosphorylates or catalyzes the transfer of a phosphate group (P0_4_), to another molecule.^15^ The transfer of a phosphate group, typically from adenosine triphosphate (ATP) to a specific amino acid residue, is central to cell cycle regulation as well as to the activity of several oncogenic signaling pathways. When the activities of regulatory kinases within the cell cycle or key biochemical pathways are altered by deactivation or gene mutation, the downstream effects can include cancer initiation and progression.

The human genome encodes over 500 kinases that mainly phosphorylate four of the 20 amino acids: serine, threonine, tyrosine, and histidine.^16^ Kinase phosphorylation can rapidly and reversibly alter the conformational shape of a protein substrate and convert it from an inactive to an active state or the reverse.^17^ Serine-threonine protein kinases add a phosphate to the hydroxyl (OH) group of either serine or threonine, while tyrosine protein kinases phosphorylate proteins at a tyrosine residue. Dual-specificity kinases have the ability to add a phosphate group to serine, threonine, as well as tyrosine. Cyclin-dependent kinases are serine-threonine kinases that regulate the integrity of the cell cycle. Tyrosine kinases are often the proto-oncogenes that can become driver components of oncogenic signaling pathways and therefore have become candidate antineoplastic drug targets.

TKIs are a class of antineoplastic drugs that inhibit the activity of tyrosine kinases present as both receptor and non-receptor (cytoplasmic) enzymes that are pivotal in promoting oncogenesis by driving signal transduction pathways. By disrupting the activity of specific tyrosine kinases, aberrant signaling can be blunted or stopped and lead to cancer cell inhibition. One group of TKIs are small-molecule oral agents that bind to or interfere with the function of the ATP-binding domain (motif) within the kinase molecule, thereby halting phosphorylation and subsequent pathway signaling.^18^ Similarities in the structure of the ATP-binding motif allows for multi-targeted TKIs to inhibit more than one type of tyrosine kinase. These small-molecule agents are named using the suffix “-itinib” at the end of the generic name. Monoclonal antibody inhibition offers another strategy to interrupt signaling by blocking the activity of tyrosine kinases that are incorporated into cell surface receptors. The U.S Food and Drug Administration (FDA) approved indication for each TKI is not only specific to cancer type and stage, but may also require the detection of defined tumor genetic alterations, referred to as biomarkers, using recommended molecular assays.^19^

## Receptor tyrosine kinases

RTKs belong to several kinase families that function as both receptors and enzymes.^14^ RTKs occur as transmembrane, polypeptide, cell surface receptors that receive and transduce signals from the extracellular to the intracellular environment by triggering a series of downstream phosphorylation events. Structurally RTKs are composed of an extracellular ligand-binding region, a transmembrane domain, an auto-inhibitory juxtamembrane region, and an intracellular catalytic tail containing tyrosine kinases. Most RTKs become activated by binding to a signaling molecule ligand at the cell surface, such as a growth factor or a peptide hormone. Ligand binding typically initiates dimerization or pairing between two neighboring receptors through cross-phosphorylation of the tyrosine molecules contained within the catalytic domain of the receptor that extends into the cell cytoplasm. Next a cascade of phosphorylation events sets forth as the tyrosine moieties interact or “dock” with molecules, including other kinases within the cytoplasm, that interface with one or more signaling pathways. Ultimately, messaging is relayed into the nucleus of the cell where it can initiate the transcription of genes controlling complex cellular processes including cell growth, differentiation, proliferation, and survival.

RTK induced oncogenesis can result from gain-of-function mutations in regions of genes that encode for the structural, regulatory, and kinase components of the receptors. Genetic alterations in RTK genes can result in receptors lacking the auto-inhibitory regulation of kinase activity that can lead to dysregulated signaling and place oncogenic pathways into a state of unchecked overdrive. Gene alteration can also produce receptors that are constitutively active, meaning they can signal independent of ligand binding.

Changes in RTK activity can also be caused by other genetic events such as structural variation. For example, a rearrangement or change in the position of a gene within a chromosome can change the structure of an RTK and its’ activity. Increased RTK signaling can also result from overexpression of the wild-type or mutated receptors on the cell surface. Termed gene amplification, this increase in the number of receptors is caused by a copy number gain resulting in the expression of multiple copies of the gene that encodes the RTK.

The Pharmacogenomics Knowledge Base has designated a category of cancer cell genes as Very Important Pharmacogenes (VIPs) Cancer Genome, because they impact tumor pharmacogenomics and offer current and future biomarker drug targets.^20^ The molecular testing or profiling of cancer genomes for VIP gene variants as well as other diagnostic biomarkers is often required to select treatment, predict response, and assess drug resistance. Among these cancer VIPs are several genes that encode RTKs. The cancer pharmacogenes discussed in the next sections encode RTKs that exemplify the link between gene variation, the oncogenic process, and the basis of targeted approaches to treatment.

## The anaplastic lymphoma receptor tyrosine kinase (*ALK*) gene

Located on Chromosome 2, the anaplastic lymphoma receptor tyrosine kinase (*ALK*) proto-oncogene encodes for the ALK receptor tyrosine kinase (ALK-RTK). Mainly inactive in adults, ALK-RTKs are normally expressed and most active in early development where they function to regulate the proliferation of nerve cells.^21^*ALK* gene mutations have been identified in over 16 types of cancer including non-small cell lung cancer (NSCLC), neuroblastomas, as well as lymphomas.^22^ In cancer cells, gain-of-function point mutations in the *ALK* gene and *ALK* gene amplification can cause ALK-RTKs to become constitutively active (ligand independent), switching on several downstream signaling pathways and ultimately resulting in an increase in cell proliferation and a reduction in apoptosis. Perhaps due to ongoing genomic instability and compromised DNA repair mechanisms in cancer cells, The *ALK* gene is also subject to large structural variations called chromosomal rearrangements.

A gene translocation occurs when a segment of DNA containing a gene changes position and becomes incorporated into a non-homologous chromosome, which is a chromosome of a different number. If this repositioning places all or a segment of a gene adjacent to another gene, the two can combine to create a hybrid or fusion gene. Changes in gene regulation and expression can also occur at the new locus. One of several known *ALK* fusions occurs when *ALK* translocates to Chromosome 5 and combines with the Nucleophosmin 1 (*NPM1*) gene to form the *NPM-ALK* fusion gene. The NPM-ALK fusion protein that results contains an activated ALK kinase domain that acts as an oncogenic driver of several pathways including MAPK/ERK, PI3K/Akt, JAK/STAT, and the PLC-gamma pathways.^23^ The *NPM-ALK* rearrangement is found in over 50% of anaplastic large T-cell lymphomas.

Fusion genes and chromosomal rearrangements can also occur within the same chromosome if a gene moves to a new locus, inverts 180 degrees, or both. One such fusion occurs when the *ALK* gene inverts and becomes inserted adjacent to the Echinoderm microtubule-associated protein-like 4 (*EML4*) gene also located on Chromosome 2. An oncogenic fusion gene called *EML4-ALK* forms and produces the EML4-ALK fusion protein kinase that acts as a driver mutation in approximately 3–5% of NSCLC. *EML4-ALK* fusions have also been identified in other cancer types such as metastatic colon cancer and breast cancer, suggesting the use of TKIs as part of the treatment plan to disrupt the kinase cascade in triggered oncogenic signaling.

*ALK* tyrosine kinase inhibitors (ALK-TKIs) are TKIs that block the overactivity of fusion and variant kinases produced from *ALK* mutations and *ALK* fusions. Crizotinib is a first-generation, oral, small-molecule ALK-TKI that was FDA approved in 2011 to treat cancers expressing *EML4-ALK* gene fusions. It competes with ATP at its’ binding pocket within the ALK receptor tyrosine kinase and tempers signaling activity. Because the ATP-binding site shares a similar structure in many RTKs, crizotinib is a multi-targeted TKI and has activity against other oncogenic receptors such as ROS proto-oncogene 1 (ROS1) and mesenchymal-epithelial transition factor (MET) discussed in later sections.^4^ Second-generation (e.g. ceritinib, alectinib, brigatinib, esartinib) and third-generation (e.g. lorlatinib) ALK-TKIs improve upon central nervous system drug penetration and can be used as indicated.

## Epidermal growth factor receptor (*EGFR*) gene

The epidermal growth factor receptor tyrosine kinase (EGFR-RTK) is a ligand-dependent RTK that is overexpressed and/or constitutively activate in a wide variety of cancer cell types including NSCL, breast, melanoma, colorectal, pancreatic, glioblastoma, esophageal, and head and neck cancers.^24^ The receptor is encoded by the *EGFR* proto-oncogene located on Chromosome 7, which is subject to oncogenic gene amplification as well as activating point mutations. The phenotypic result of these gene alterations is the overexpression of unregulated EGFR-RTKs exhibiting a loss of auto-inhibition of the tyrosine kinase domain that drives cell division through increased oncogenic pathway signaling. Be aware that the *EGFR* gene is also known as the human epidermal receptor 1 gene (*HER1*), as well as the erythroblastic leukemia viral oncogene receptor 1 gene (*ERBB1*) and belongs to the ERBB superfamily of RTKs.

In mammals, EGFR-RTKs are normally expressed in fibroblasts, epithelial cells, and neurons, to play a role in dermal and epidermal wound healing as well as the differentiation, maturation, and survival of neurons in the central nervous system. An EGFR-RTK is activated mainly in response to the extracellular binding of a protein ligand called the epidermal growth factor (EGF). EGF is known as a mitogen factor because by activating the EGFR-RTK, it ultimately transmits signaling into the cell nucleus to begin the process of mitosis, drive the cell cycle forward, and inhibit apoptosis.^25^ Note that EGFR-RTKs and other members of the ERBB superfamily share several additional growth factor ligands that can activate the receptors and elicit a diversity of responses within a complex network of oncogenic pathways.

In keeping with the usual activity of RTKs, EGF binding causes receptor dimerization and autophosphorylation of the intracellular tyrosine kinase domain. Dimerization not only occurs between two EGFRs forming a homodimer but can also occur between two different receptors within the ERBB superfamily to form a heterodimer. The specific heterodimer formed by the combination of EGFR and another ERBB family member is thought to be influenced by the type of ligand and produces a diversity of signaling within a network of pathways. Intracellular signaling can result in the activation of the MAPK/ERK, the PI3K/Akt, and other interfacing pathways resulting in a wide variety of oncogenic forces including mitogenic gene expression in the nucleus.

While many different activating mutations in *EGFR* can occur, specific variants serve as molecular drug targets for several small-molecule oral TKIs classified as epithelial growth factor receptor tyrosine kinase inhibitors (EGFR-TKIs). The FDA-approved indication for individual EGFR-TKIs often requires the molecular profiling of tumor DNA for the presence of variants in specific cancer cell types to predict the likelihood of response or the development of drug resistance. In NSCLC for example, these activating mutations are often located within exons 18–21, because this is the region of the *EGFR* gene that encodes the intracellular tyrosine kinase domain of the receptor.^26^ Depending upon the agent, EGFR-TKIs reversibly or irreversibly inhibit the binding of ATP within the tyrosine kinase domain and blunt receptor signaling.

One such molecular target that predicts EGFR-TKI efficacy is the presence of an *EGFR* exon 19 deletion (E19del). The deletion is caused by missing nucleotide bases within exon 19 and produces a receptor with structural changes that confer a higher binding affinity over ATP for EGFR-TKIs such as afatinib, erlotinib, gefitinib, and dacomitinib. These same EGFR-TKIs are also indicated in the presence of NSCLC cells expressing the *EGFR* exon 21 (L858R) substitution (missense) variant caused by a single nucleotide change (CTG to CGG) that encodes the substitution of an arginine (R) at codon 858 instead of the leucine (L) amino acid that is normally present and favoring drug binding.

As they proliferate NSCLC cells can acquire or intrinsically possess mutational changes that make them resistant to first- and second-generation EGFR-TKIs.^27^ One recognized mechanism of resistance is the insertion of additional base pairs in exon 20 that adds 1–7 additional amino acids to the structure of the EGFR, called exon 20 insertion mutations (*EGFR* ex20ins). Not only does this conformational change act as a driver mutation by rendering the receptor constitutively active, but also disfavors EGFR-TKI binding producing resistance. Mobocertinib is a third-generation, irreversible EGFR-TKI that maintains efficacy and specifically targets NSCLC cells expressing *EGFR* ex20ins. The monoclonal antibody antineoplastic agent amivantamab was also recently approved for NSCLC expressing *EGFR* ex20ins mutations.

Another mechanism of drug resistance is the *EGFR* exon 20 T790M (*EGFR* T790M) substitution mutation found in approximately 50% of NSCLC genomes post-exposure to EGFR-TKIs.^28^ The *EGFR* T790M substitution changes the structure of the kinase domain, allowing an increase in the affinity of ATP to its’ binding pocket and overcoming EGFR-TKI inhibition. Osimertinib is a third-generation, irreversible EGFR-TKI that maintains efficacy in the presence of the *EGFR* T790M secondary mutation, as well as selectivity to both *EGFR* exon 19 deletions and the *EGFR* exon 21 L858R sensitizing mutation.

## Human epidermal growth factor receptor 2 (*HER2/ERBB2*) gene

Another member of the ERBB superfamily of RTKs is the ligand-independent human epidermal growth factor receptor 2 (HER2-RTK), officially named the erb-b2 receptor tyrosine kinase 2 (ERBB2-RTK). The discovery in the late 1980s that over 20% of breast cancer cells overexpressed HER2-RTKs on their surfaces, ushered in a new approach to cancer treatment directed at targeting the expression of tumor biomarkers and disrupting the activity of gene products. The receptor is encoded by the proto-oncogene *HER2/ERBB2* located on Chromosome 17 that can undergo massive copy number gains of as much as 25–50 gene copies, resulting in the expression of millions of HER2-RTKs on cancer cell surfaces.^29^*HER2/ERBB2* gene amplification is recognized as an oncogenic driver in breast, ovarian, gastric, gastroesophageal, endometrium, bladder, lung, colon, and head and neck cancer cells.

Trans-autophosphorylation of the intracellular tyrosine kinase domain of a HER2-RTK is necessary for receptor activation and occurs through homodimerization between HER2-RTKs in close proximity, as well as through heterodimerization with other family members such as an EGFR-RTK. Overexpression of HER2-RTKs makes dimerization much more likely and results in the oncogenic overdrive of several downstream mitogenic pathways including the MAPK/ERK and the PI3K/Akt signaling cascades. Inhibiting the autophosphorylation of the tyrosine kinase domain and the ensuing receptor signaling is achieved through the use of monoclonal antibodies and TKIs.

Approved by the FDA in 1998, trastuzumab was one of the first anti-cancer monoclonal antibody drugs developed to impede HER2-RTK dimerization and interrupt signaling. As the extracellular region of a HER2-RTK lacks a ligand-binding portion, monoclonal antibody drugs interrupt signaling by binding to the juxtamembrane region that links the transmembrane to the intracellular tyrosine kinase domain.^30^ The structure of the juxtamembrane region is genetically “conserved” or similar across many different RTKs and functions to regulate and activate dimerization of the tyrosine kinase domain. Trastuzumab and the recently approved margetuximab block receptor activation by attaching to an area of the juxtamembrane region. Pertuzumab attaches and inhibits at a different juxtamembrane binding site and is often used in combination with trastuzumab to provide synergy against heterodimerization.

While monoclonal antibody inhibition is the cornerstone of HER2-positive cancer therapy, directly blocking the ATP phosphorylation of the intracellular tyrosine kinase domain and interrupting downstream signaling can also be achieved through the use of several oral, small-molecule TKIs. Afatinib, lapatinib, and neratinib are referred to as multi-targeted TKIs because they block the ATP-binding pocket of both HER2-RTKs and EFGR-RTKs, prevent autophosphorylation, and halt downstream signaling.

## The KIT proto-oncogene, receptor tyrosine kinase (*KIT, c-KIT*) gene

The KIT proto-oncogene receptor tyrosine kinase (KIT-RTK) is a ligand-dependent receptor that begins signal transduction in response to the extracellular binding of a cytokine called stem cell factor (SCF).^31^ KIT-RTKs are normally expressed in germ cells, testicular germ cells, hematopoietic stem cells, mast cells, melanocytes, and gastrointestinal tract interstitial cells of Cajal. Along with the platelet-derived growth factor receptors, the KIT-RTK is a member of the tyrosine kinase Type III family of receptors that function to prompt nuclear gene transcription that supports cell growth, development, and migration. The signal transduction elicited by KIT-RTKs intersects several of the aforementioned oncogenic pathways including MAPK/ERK, PIK3/Akt, JAK/STAT, and PLC-gamma.

The *KIT* gene is encoded on Chromosome 4 and can be subject to oncogenic amplification as well as gain-of-function alterations including point mutations, deletions, and insertions. Oncogenic activating mutations of the *KIT* gene are known to occur most commonly at multiple loci within exons 9, 11, 13, and 17. *KIT* gene alterations often happen in combination, resulting in the overproduction of mutated, constitutively active KIT-RTKs, which transduce signals without the control of SCF ligand binding. Cancers associated with *KIT* alterations include gastrointestinal stromal tumors (GISTs), melanomas, lung cancer, and seminoma testicular cancer.^32^

GISTs are sarcomas that arise from the mesenchymal cells of the gastrointestinal tract including the widely distributed, interstitial cells of Cajal, which function as pacemakers for intestinal motility. *KIT* gene gain-of-function mutations occur in 80% of GIST cells and are considered the main oncogenic drivers of proliferation and metastasis. The most common activating mutations occur within exon 11, which encodes the juxtamembrane domain of the KIT-RTK. These *KIT* gene mutations result in the production of KIT-RTKs that dimerizes independent of SCF binding with unregulated activation of the tyrosine kinase domain, and subsequent oncogenic signaling.^33^ Exon 9 alterations are found in approximately 10% of GISTs and are typically located in the small intestine. These mutations result from duplications of nucleotide bases and codons within exon 9 that change the structure of a regulatory region at the membrane surface and produce KIT-RTKs that dimerize more readily. Exon 9 mutations also occur in some testicular Seminole cancers.

GISTs and other cancers expressing un-mutated, wild-type KIT-RTKs, as well as those possessing exon 11, exon 9, and other less common activating mutations, can be inhibited with the KIT proto-oncogene receptor tyrosine kinases (KIT-TKIs) imatinib and ripretinib. KIT-TKIs interrupt receptor signaling to downstream oncogenic pathways by attaching to sites either within or in close proximity to the ATP-binding pockets of the tyrosine kinase domain. Imatinib binding changes the conformation of the pocket so that ATP cannot access it and tyrosine phosphorylation does not occur. When KIT exon 9 mutations are present, a higher dose of imatinib is required to overcome the competitive inhibition with ATP and suppress signaling.

Resistance to imatinib and other small-molecule KIT-TKIs is the consequence of genomic instability and an ongoing oncogenic process where tumor cells continue to acquire secondary mutations that allow them to adapt and survive. Imatinib competitive inhibition is overcome by secondary mutations causing changes in the pocket structure that limit imatinib binding in favor of ATP. Ripretinib is a next-generation KIT-TKI that is indicated for advanced GISTs that have acquired secondary resistance mutations.^34^ Ripretinib circumvents resistance through interference with an adjacent polypeptide region called the activation loop. Ripretinib attachment to the activation loop switches the conformation of the kinase pocket to a “closed” state that blocks ATP phosphorylation. It has broad activity against KIT-RTKs harboring both activating primary and secondary mutations occurring in many different *KIT* exons.

## Mesenchymal-epithelial transition proto-oncogene, receptor tyrosine kinase (*MET*) gene:

The mesenchymal-epithelial transition factor (*MET*) gene is a proto-oncogene that encodes for the ligand-dependent MET receptor tyrosine kinase (MET-RTK). MET-RTK is another member of the ERBB superfamily of receptors and is activated by binding hepatocyte growth factor (HGF), which is a cytokine secreted by mesenchymal cells. HGF binding initiates a downstream signaling cascade of several interwoven pathways including MAPK/ERK, PIK3/Akt, and others that promote mitogenesis, cell motility, invasiveness, and angiogenesis.^35^ MET-RTK signaling is most active during embryonic development but can be activated in children and adults when needed for wound healing and tissue regeneration. Structural variation as well as amplification of the *MET* gene has been identified in the genomes of many different cancer types including NSCLC, papillary renal cell carcinoma, breast, colon, hepatocellular carcinoma, and various head and neck cancers. Aberrant, excess signaling by MET-RTK can drive tumorigenesis and is believed to play a role in the ability of cancer cells to invade surrounding tissue, spread, and metastasize.

*MET* gene oncogenic alterations can result from point mutations, amplification (*MET*-amplified), and a specific gene mutation called *MET* exon 14 skipping.^36^ Exon skipping is a phenomenon that occurs early in the process of transcription as pre-messenger RNA copies the nucleotide bases making up both the exons and the introns of the gene. Next, the non-coding intron copies are spliced or cut out of the transcript leaving the protein-coding exons to form messenger RNA. If a misalignment or other flaw is detected within an exon, it is also spliced and that portion of the code is not brought forward to be translated into the protein structure. Typically exon skipping results in a non-functional protein but in the case of the *MET* gene, eliminating exon 14 results in the production of hyperactive MET-RTKs that are missing the juxtamembrane region needed for negative regulation of kinase activity. Receptors harboring *MET*ex14 mutations can be inhibited with specific TKIs and monoclonal antibody antineoplastic agents.

Both TKIs and monoclonal antibody antineoplastic agents can be used to halt downstream signaling by MET-RTKs that possess *MET* exon 14 mutations (*MET*ex14). The TKIs crizotinib, capmatinib, and tepotinib are indicated for the treatment of NSCLC that is tumor or blood specimen positive for the *MET*ex14 mutation. If *MET*ex14 mutations are found concurrent with ALK rearrangements in NSCLC, the multi-targeted crizotinib can be particularly useful because it has shown effectiveness when each is present. The aforementioned EGFR-RTK monoclonal antibody amivantamab also has the ability to inhibit MET-RTKs expressing the *MET*ex14 skipping mutation.

## ROS proto-oncogene 1, receptor tyrosine kinase (*ROS1*) gene

The *ROS1* gene located on Chromosome 6 encodes the ROS proto-oncogene 1, receptor tyrosine kinase (ROS1-RTK), which belongs to a subclass of the insulin receptor family.^37^ Although the normal function of ROS1-RTKs is largely unknown, genetically altered receptors are present in approximately 4% of all cancers including melanomas, lung, colon, endometrial, ductal breast, and many other cancer types.^38^ The *ROS1* gene is subject to multiple oncogenic gene fusions that create proteins containing the catalytic tyrosine kinase domain of the receptor without the extracellular components that control signaling.

The tyrosine kinase domain of the ROS1-TKI shares 70% of its structure with that of the ALK-RTK and activates many of the same oncogenic pathways including the MAPK/ERK, PI3K/Akt, and JAK/STAT. It follows then that many of the same drugs that inhibit ALK-RTKs are also effective in inhibiting ROS1-TKI signaling.

## Future direction

Current and future advancements in tumor molecular profiling, as well as the bioinformatics techniques needed to interpret results, are creating new paradigms for the selection of cancer treatments that are precisely targeted to each tumor's unique mutational profile.^39^ Molecular profiling is a complement to the traditional histopathological exam and can help identify the best areas of a tumor tissue sample to prepare for analysis. Efficiencies and cost reductions arising from the use of next-generation sequencing (NGS) make the sequencing of tumor DNA and RNA transcripts as well as copy number analysis, clinically feasible. Identification of oncogenic fusions, translocations, and mutations that are targetable with antineoplastic agents including TKIs helps insure that the most effective treatments are precisely chosen.

Perhaps most promising for the translation of genomic-guided drug selection is the development and availability of minimally invasive molecular diagnostic tests called liquid biopsies. As tumors grow, they shed cells and their nucleic acids into the circulation and other body fluids. Through peripheral blood/plasma sampling, circulating tumor DNA (ctDNA) and tumor RNA can be captured and sequenced to identify important therapeutic biomarkers. Serial liquid biopsies can be examined throughout the disease course and used to assess treatment response and identify the emergence of resistance or recurrence. Most hopeful is the possibility of using liquid biopsies to screen people for undiscovered cancers at an early or pre-symptomatic stage.

The challenge for pharmacists is to stay updated about the quickly expanding, genetic-based mechanisms for cancer therapies. It is hoped that this review demonstrates some of the basic concepts underlying biomarker-directed use of TKIs and that it will also enlighten the understanding of future genetic-based therapies. Learning about genomic-guided drug therapies not only is professionally fulfilling but presents opportunities for pharmacists to optimize and personalize drug therapy.
